# Oncological outcome and immune-checkpoint-blockade-induced toxicities in patients with cervical cancer – a Norwegian real-world cohort

**DOI:** 10.2340/1651-226X.2026.45737

**Published:** 2026-06-01

**Authors:** Katharina Bischof, Keira Matallah Fredriksen, Margrethe Bjørnstad, Mari Bunkholt Elstrand, Brynhildur Eyjolfsdottir, Alda Birgisdottir, Elisabeth Lillo, Guro Aune, Kristina Lindemann

**Affiliations:** a Department of Surgical Oncology, Section for Gynaecological Oncology, The Norwegian Radium Hospital, Oslo University Hospital, Oslo, Norway; b Department of Cancer Immunology, Faculty of Medicine, Institute of Clinical Medicine, University of Oslo, Oslo, Norway; c Department of Clinical and Molecular Medicine, Faculty of Medicine, Norwegian University of Science and Technology, Trondheim, Norway; d Department of Surgical Oncology, Section for Research and Clinical Studies, The Norwegian Radium Hospital, Oslo University Hospital, Oslo, Norway; e Department of Gynaecological Oncology, St. Olavs Hospital, Trondheim University Hospital, Trondheim, Norway; f Faculty of Medicine, Institute of Clinical Medicine, University of Oslo, Oslo, Norway

**Keywords:** Uterine cervical neoplasms, gynaecologic cancer, immune checkpoint inhibitors, real-world data

## Abstract

**Background and purpose:**

The introduction of immune checkpoint inhibitors (ICIs) into the treatment of patients with metastatic or recurrent cervical cancer has been shown to significantly prolong survival. In this study, we examined the clinical outcomes and tolerability of treatment with ICIs in patients with metastatic or recurrent cervical cancer in a real-world setting in Norway.

**Patient/material and methods:**

This retrospective cohort study included patients treated with an ICI in combination with chemotherapy or as single agent at Oslo University Hospital between 2016 and 2024. The primary oncological endpoint was progression-free survival (PFS). Secondary endpoints include overall survival (OS) as well as tolerability.

**Results:**

We included 57 patients with a median age of 53 years and a median follow-up of 15.2 months. Thirty-five patients were treated with an ICI in combination with chemotherapy (cohort 1), and or an ICI alone (*n* = 22) (cohort 2). Forty-six patients (81%) were treated for recurrent disease. In cohort 1, the median PFS was 12.4 months (95% CI: 9.0–15.7), and the median OS was 27.5 months (95% CI: 18.0–37.1). In cohort 2, the median PFS was 3.7 months (95% CI: 2.4–5.0), and the median OS was 9.3 months (95% CI: 4.3–14.4). Nine patients (16%) discontinued treatment due to toxicity.

**Interpretation:**

Our real-world data on the use of ICIs alone or in combination showed antitumour efficacy comparable to that reported in clinical studies in patients with advanced or recurrent cervical cancer. Our discontinuation rate highlights that toxicity management and mitigation are paramount when novel drugs are introduced in clinical algorithms.

## Introduction

Cervical cancer remains a significant global health challenge, ranking as the fourth most common cancer among women worldwide. According to the World Health Organization, over 600,000 new cases and approximately 342,000 deaths were reported in 2020 alone [1]. In Norway, the incidence of cervical cancer has markedly declined due to the introduction of organised, high-coverage screening programs and widespread Human Papilloma Virus (HPV) vaccination [2]. However, patients diagnosed with primary metastatic or recurrent cervical cancer still face poor prognoses and have limited therapeutic options.

Immune checkpoint blockade has emerged as a promising treatment modality for various cancers, including cervical cancer. In 2019, the KEYNOTE-158 trial highlighted the activity of pembrolizumab, an immune checkpoint inhibitor (ICI), blocking the cell surface receptor programmed death-1 (PD-1), as a single agent in recurrent or metastatic cervical cancer, showing an objective response rate (ORR) of 14.3% in a biomarker-unselected population. 17% of patients with tumours showing expression of programmed death ligand 1 (PD-L1) had objective response to the treatment [3]. The KEYNOTE-826 trial later demonstrated that the addition of pembrolizumab to standard chemotherapy ± bevacizumab significantly prolonged progression-free survival (PFS) from 8.2 to 10.5 months and overall survival (OS) from 16.5 to 28.6 months in patients with persistent, recurrent, or metastatic cervical cancer with PD-L1-positive tumours [4]. Another phase 3 trial, testing the efficacy of cemiplimab, a PD-1 inhibitor, monotherapy in patients with relapsed cervical cancer after platinum-containing chemotherapy, reported an ORR of 16.4% in all patients with an enhanced efficacy in patients with PD-L1 positive tumours and with durable responses observed in a subset of patients [5]. Comparable results were reached in the ENGOT-Cx10/BEATcc trial, where the PD-L1 checkpoint inhibitor atezolizumab was added to standard treatment including bevacizumab plus platinum-based chemotherapy in the same patient population [6]. In this study, the median PFS was extended by 3.3 months, while the median OS was prolonged by 9.3 months from 22.8 months in the control group to 32.1 months in the ICI treated population.

Real-world studies have provided valuable, additional evidence supporting the use of ICIs. Retrospective analyses of patients treated outside clinical trials have added to the current knowledge, corroborating response rates and survival outcomes derived in controlled settings. The Korean multi-centre retrospective study (KGOG1041) evaluated the pembrolizumab monotherapy in 117 patients with recurrent or persistent cervical cancer [7]. The study found that pembrolizumab was effective in achieving durable responses in a subset of patients, with outcomes consistent with those reported in earlier clinical trials. Three (2.6%) and eight patients (6.8%) achieved a complete response (CR) or partial response (PR), respectively, resulting in a median ORR of 9.4% (95% CI, 4.8–16.2). Furuya et al. recently published a comparable dataset reporting a total of 23% CR after treatment with pembrolizumab [8]. Another real-world study from Italy, MITO44, investigated efficacy and safety of cemiplimab in advanced cervical cancer. The authors reported safety and efficacy of the drug that were compatible with the results from the phase-3-trial EMPOWER-CERVICAL-1 [9].

### Aim

The aim of the present study was to generate data on the efficacy and toxicity of treatment with ICIs for patients with primary metastatic, persistent, or recurrent cervical cancer in an unselected Norwegian patient cohort.

## Patients/material and methods

In this single-centre, retrospective study, women over 18 years with primary metastatic or recurrent cervical cancer, was included. Patients were treated with either ICI plus chemotherapy and/or bevacizumab (cohort 1) or an ICI alone after progression on platinum containing chemotherapy (cohort 2). The study population received treatment between November 2016 and October 2024 at the Department of Surgical Oncology, Section for Gynaecological Oncology, Oslo University Hospital, Norway and was identified using the institutional quality registry. While treatment algorithms were supervised by experts at Oslo University Hospital, treatment for a substantial part of the patients was administered at secondary centres throughout South-Eastern Norway. All response evaluations were performed at Oslo University Hospital by trained staff. The study population consists of two defined treatment settings differing in prognosis. Therefore, for the analysis of oncological outcome, cohorts 1 and 2 were analysed separately.

Clinical baseline characteristics as well as information on oncological outcome and safety were collected from the institutional quality registry and from the patient electronic records. During treatment with ICIs, patients were monitored closely with pre-treatment blood samples including complete blood count, assessment of renal function and thyroid function and glucose levels and cortisol before each treatment cycle. Upon clinical suspicion of heart failure or symptoms of pancreatitis, cholangitis, or hepatitis, biochemical analyses were extended to liver function parameters, amylase and pro-BNP. Adverse events (AEs) of grade 3–5 and immune-checkpoint-related adverse events (irAEs) of any grade were retrospectively graded based on documentation in the patient’s electronic records and coded according to National Cancer Institute Common Terminology Criteria for Adverse Events (CTCAE) version 5.0 [10] and the ESMO Clinical Practice Guideline – Management of Toxicities from Immunotherapy [11]. The primary endpoint was PFS defined as time from first dose of ICI to investigator assessed progression of disease (preferably by CT). Secondary endpoints included OS, defined as time from start of ICI treatment to death of all cause. Safety and tolerability parameters, assessed as prevalence of and reasons for dose discontinuation and reduction, were investigated together with duration of ICI treatment and toxicity. Individual survival data were available through linkage to Statistics Norway.

The study was approved by the Regional Committees for Medical Research Ethics – South- East Norway (Reference no. 536885) and by the data protection officer at Oslo University Hospital. Only patients who had earlier provided informed consent were included in the present study. A waiver was given for patients who had deceased.

### Statistical analysis

Patient characteristics were summarised with frequencies and percentages for categorical variables and median/range for continuous variables. Pearson’s chi-squared test or Fisher’s exact test for categorical variables was performed when comparing differences between two independent groups. Two-sided *p*-values of < 0.05 were considered statistically significant. PD-L1 status was assessed with combined positive score (CPS) defined as the number of PD-L1staining cells (tumour cells, lymphocytes and macrophages) divided by the total number of viable tumour cells, multiplied by 100 [12]. Tumours were classified as PD-L1 positive if the CPS was ≥ 1.

Clinical outcome data as PFS and OS were analysed using the univariate Kaplan–Meier method, with start of follow-up defined as date of first treatment. Patients without an event at last follow-up on September 30, 2024, were censored at this time point. Death from the disease under study was used as a surrogate for progression where not formally recorded; patients dying from unknown causes (*n* = 5) were censored at date of death for PFS. Progression was assessed clinically before each treatment cycle, and by imaging every 3 months or more frequently if indicated. Analyses were performed in SPSS (version 27) and Stata (version 19.5).

## Results

**Figure 1:**
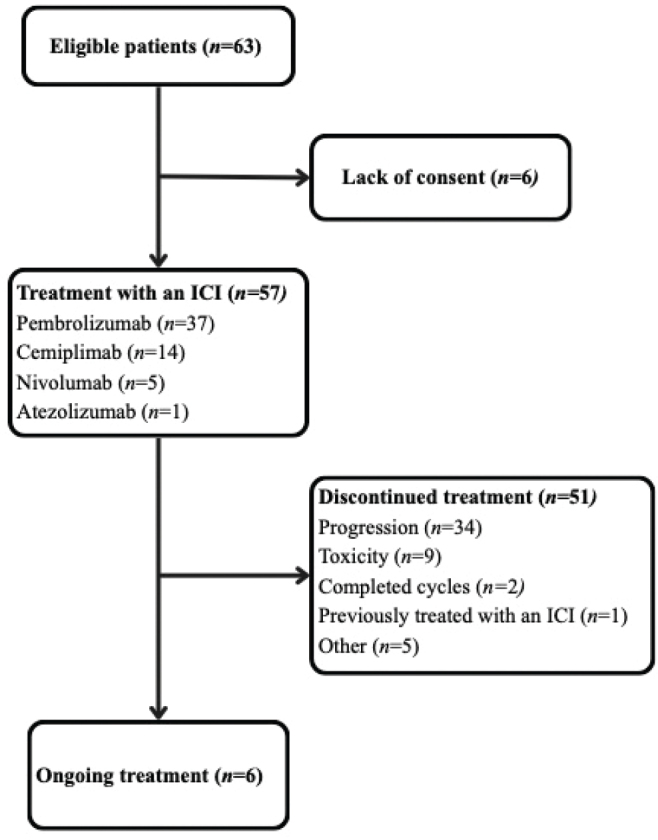
Displays the process of identifying the patient population at a single-centre and informs about the status of treatment with ICIs at cut-off. ICI: immune checkpoint inhibitors.

Of the 63 patients who were identified in the institutional registry, 57 patients consented to participate (Figure 1). Thirty-five (61.4%) of the patients had primary metastatic/recurrent disease and were treated with an ICI-chemotherapy ± bevacizumab combination (cohort 1), whereas 22 (38.6%) had recurrent disease and were treated with a single agent ICI (cohort 2). PD-L1 score was assessed in a total of 48 patients (84%), confirming CPS > 1 in all samples. A greater proportion of patients in cohort 1 exhibited a CPS score > 1 compared to those in cohort 2, with 34 (97%) and 14 (64%) positive samples, respectively.

Most patients (61%, *n* = 35) were diagnosed with squamous cell carcinoma and presented with an Eastern Cooperative Oncology Group (ECOG) Performance status of 0 (44%, *n =* 25). Four patients (7%) showed reduced performance statuses (ECOG 3) at baseline, overrepresented (*n* = 3) in cohort 2. Cohort 1 consisted of 11 patients (31%) presenting with primary metastatic cervical cancer, while the remaining 24 patients were treated for relapse (69%). Sixteen women (28%) had received ≥ 3 prior lines of therapy before ICI treatment as illustrated in Table 1.

**Table 1:** Summary of clinical baseline characteristics.

Characteristics	Combination *n* = 35 (61.4%)	Single agent *n* = 22 (38.6%)
**Age, years**		
Median (range)	52 (27–83)	55.5 (33–78)
**ECOG**		
0	19 (54%)	6 (27%)
1	13 (37%)	9 (41%)
2	2 (6%)	4 (18%)
3	1 (3%)	3 (14%)
**FIGO stage**		
I	8 (23%)	5 (23%)
II	4 (11%)	6 (27%)
III	7 (20%)	5 (23%)
IV	15 (43%)	5 (23%)
Unknown	1 (3%)	1 (5%)
**Disease status**		
Primary metastatic	11 (31%)	0
Recurrent	24 (69%)	22 (100%)
PDL score*		
> 1	34 (97%)	14 (64%)
Not assessed	1 (3%)	8 (35%)
**Histology**		
Adeno(squamous) carcinoma	9 (26%)	7 (32%)
Squamous-cell carcinoma	21 (60%)	14 (64%)
Other	5 (14%)	1 (5%)
**Prior lines of therapy**		
0	11 (31%)	0
1	16 (46%)	3 (14%)
2	7 (20%)	4 (18%)
≥ 3	1 (3%)	15 (68%)

FIGO: Fédération Internationale de Gynécologie et d’Obstétrique; PDL: Programmed Death-Ligand 1; ECOG: Eastern Cooperative Oncology Group

* PD-L1 score was measured with CPS (Combined Positive Score)

Among patients that received combination treatment with ICIs, 71% (*n* = 25) also received concomitant vascular-endothelial growth factor targeting drugs (bevacizumab). A total of 37 (65%) patients were treated with pembrolizumab, mainly in cohort 1 (*n* = 33), while cemiplimab was more routinely administered in cohort 2 in 13 cases. Five patients received nivolumab for their relapses, and one case was treated with atezolizumab. For the whole population, median number of ICI cycles were 11 (1–41), with 15 (1–41) and 6 (1–36) in cohorts 1 and 2, respectively, see Table 2.

**Table 2:** Part A: displays the use of individual ICIs and vascular endothelial growth factor blockade as well as number of ICI cycles. Part B: Oncological outcome parameters PFS and OS and best response to treatment.

Part A: Treatment	Cohort 1 (*n* = 35)	Cohort 2 (*n* = 22)
**Immunotherapy drug**		
Pembrolizumab	33 (94%)	4 (18%)
Cemiplimab	1 (3%)	13 (59%)
Nivolumab	0	5 (23%)
Atezolizumab	1 (3%)	0
**Cycles**		
Median (range)	15 (1–41)	6 (1–36)
**Concomitant targeted therapy**		
Bevacizumab	25 (71%)	0
**Concomitant chemotherapy**		
Carboplatin/Cisplatin	33 (58%)	0
Paclitaxel/Docetaxel	31 (54%)	0
Etoposide	1 (2%)	0
Topotecan	1 (2%)	0
**Part B: Outcome**		
**Progression free survival (months)**		
Median	12.39	3.71
95% CI	9.04–15.72	2.44–4.99
**Overall survival (months)**		
Median	27.53	9.33
95% CI	18–37.05	4.3–14.35
**Follow-up time (months)**		
Median (range)	18.0 (5.2–47.9)	9.38 (1.2–93.3)
**Best response**		
Complete response	1 (2.9%)	1 (4.5%)
Partial response	13 (37.1%)	4 (18.1%)
Stable disease	9 (25.7%)	4 (18.1%)
Progression	6 (17.1%)	10 (45.5%)
Not evaluated	6 (17.1%)	3 (13.6%)

Note that total amount and percentage of chemotherapy and targeted therapy exceed the total in cohort 1, due to patients who received several drugs in their treatment regimen. ICI: immune checkpoint inhibitors; PFS: progression-free survival; OS: overall survival.

The median follow-up time was 18 months (range 5.2–27.9.0) in cohort 1 and 9.38 months (range 1.2–93.3) in cohort 2. Across the entire study population, after a median follow-up of 15.2 months, 51 patients (89.5%) had discontinued treatment and 34 (59.6%) of those had progressed. Additionally, one patient discontinued ICI treatment after one cycle only due to prior progression on immunotherapy.

**Figure 2:**
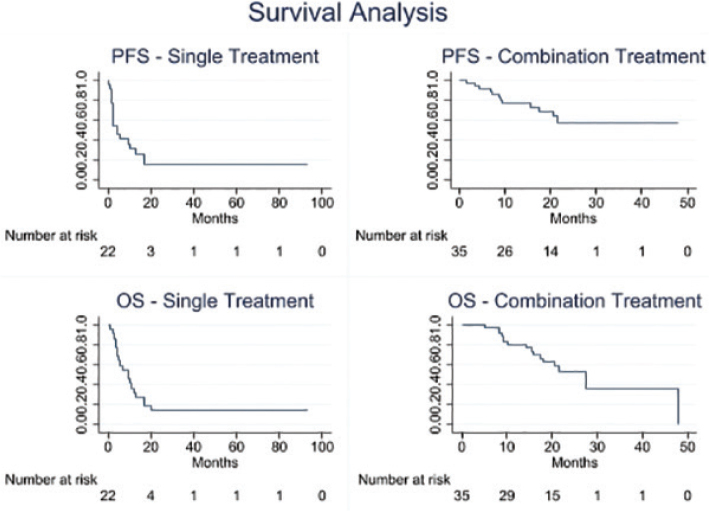
Kaplan–Meier estimates of progression-free survival (PFS) and overall survival (OS) in months depicted for individual cohorts.

In cohort 1, one patient (2.9%) achieved a CR, while 13 patients (37.1%) exhibited a PR. Additionally, nine patients (25.7%) reached stable disease (SD), while six patients (17.1%) demonstrated disease progression (PD). In cohort 2, one patient (4.5%) achieved CR, four patients (18.1%) exhibited PR and SD, respectively, and 10 patients (45.5%) demonstrated PD. Nine patients (15.8%) in total, six in cohort 1 (17.1%) and three in cohort 2 (13.6%) had not yet been evaluated for response at the time of data cut off. Overall, the median PFS was 8.9 months (95% CI: 5.0–12.8), while the median OS was 17.1 months (95% CI: 14.1–20.1). In cohort 1, the median PFS was 12.4 months (95% CI: 9.0–15.7), and the median OS was 27.5 months (95% CI: 18.0–37.1). Patients enrolled in cohort 2 showed a median PFS of 3.7 months (95% CI: 2.4–5.0) and a median OS of 9.3 months (95% CI: 4.3–14.4) (Table 2 and Figure 2).

A total of 50 patients experienced an AE of any grade. The majority of the documented AEs were severe (grade 3), three patients experienced an AE of grade 4, whereas no grade 5 toxicity has been reported. AEs were reported in 17/22 (77%) patients in the single treatment group and 33/35 (94%) in the combination group. The median number of AE episodes per patient was 2 (range 1–10) and 4.5 (range 1–56) respectively. Dose interruptions due to AEs occurred in seven (31.8%) and 16 (45.7%) patients in the single and combination groups, respectively. Treatment withdrawal due to AEs was required in three (13.6%) and six (17.1%) patients respectively. Scheduled treatment was postponed due to AEs in 22 (39%) patients, while treatment had to be withdrawn in nine (16%) cases. A comprehensive overview over AEs along with their respective grades and handling of high-grade toxicities is presented in Table 3. A total of seven patients did not develop documented toxicities under ICI treatment. Supplementary Table 1 displays all documented AEs in detail, and irAEs are displayed in Supplementary Table 2.

**Table 3:** Overview over all documented AEs together with dose interruptions and withdrawal and medical management of high-grade toxicities.

Grade	Cohort 1 (*n* = 35)	Cohort 2 (*n* = 22)
1	3 (9%)	4 (18%)
2	9 (26%)	3 (14%)
3	18 (51%)	10 (46%)
4	3 (9%)	0
No AE	2 (6%)	5 (23%)
**Action taken**		
Drug withdrawal*	6 (17%)	3 (14%)
Dose interruption*	14 (40%)	7 (32%)
Treatment of high-grade AEs**		
Steroids	12 (34%)	6 (27%)
Antihistamines	4 (11%)	1 (5%)
Monoclonal antibodies (TNFα)	2 (6%)	0
Endocrine replacement	6 (17%)	2 (9%)

* Note that individual patients can have experienced dose interruption and withdrawal;

** Displaying percentage relative to all registered toxicities in Supplementary Table 1 (*n* = 259). AE: adverse events; TNF: Tumor necrosis factor

## Discussion and conclusion

This single-centre, retrospective study evaluated real-world outcomes of patients treated with ICIs – either as monotherapy and in combination regimens – for advanced or recurrent cervical cancer. Despite our patient population presenting with less favourable baseline characteristics compared to those enrolled in pivotal clinical trials, we report efficacy outcomes in terms of PFS and OS that are broadly comparable to those published in practice-changing phase 3 studies [4–6]. Our patients experienced a notably higher rate of AEs, resulting in delayed ICI dosing in 39% of cases and treatment discontinuation in 16%. Among patients treated with ICI-based combination therapy (cohort 1), we observed a median PFS of 12.4 months, and a median OS of 27.5 months, which are comparable to the outcomes reported in the KEYNOTE 826 and ENGOT-Cx10/BEATcc trials [4, 6]. The combination backbone used in cohort 1 reflects established practice, with bevacizumab having demonstrated a significant improvement in OS when added to platinum-based chemotherapy prior to the immunotherapy era [13, 14]. The numerically shorter estimates in our cohort may, at least in part, reflect differences in patient selection, the more stringent inclusion criteria applied in phase 3 trials and the mandatory use of bevacizumab in the ENGOT-Cx10/BEATcc study. The rate of discontinuation due to treatment-related toxicity was 15% in the ENGOT-Cx10/BEATcc study and 5.9% in the KEYNOTE 826 study. Our overall discontinuation rate of 16% is comparable, bearing in mind that our series additionally encompassed patients receiving monotherapy following progression on platinum-based drugs. Patients in cohort 2, treated with ICI monotherapy, experienced disease progression after a median PFS of 3.7 months and a median OS of 9.3 months. This reflects the inherently poor prognosis patients progressing after platinum-based chemotherapy.

Our real-world findings can be contextualised against data from several other retrospective real-world series: the KGOG1041 study and the recently published series from Furuya et al, both comprising patients treated exclusively with pembrolizumab monotherapy and the MITO44 study – as a multicentre series – reporting on patients treated with cemiplimab in an early access programme [7–9].

The CR rate of 23% in the pembrolizumab monotherapy population, published in late 2025 [8], is notably higher than that observed in both our monotherapy cohort and in KGOG1041, and the median OS of 47.5 months is substantially more favourable than any other monotherapy real-world series to date. These striking differences likely reflect important patient selection effects: the study demonstrated that patients with an ECOG performance status greater than 1 faced a sixfold increased hazard of death compared to those with a status of 0 or 1. In our cohort 2, a higher proportion of patients presented with reduced performance status at baseline, which may in part explain the more modest outcomes we observed. Toxicity in that series also appeared considerably more manageable, with only 7% of AEs classified as grade 3 or higher – markedly lower than the 51% of grade 3 or higher AEs observed in our study.

Baseline characteristics varied to some extent across all these real-world studies. Our patients were more heavily pre-treated compared to those in KGOG1041, although we included a comparable proportion of patients with ECOG ≥ 2, while MITO44 restricted enrolment to ≤ ECOG 2. PD-L1 status was not rigorously assessed in either of the comparator studies. The disease control rate observed in KGOG1041 (33%) was lower than that reported in our cohort and the MITO44 study [7, 9]. The median PFS in our monotherapy cohort and in KGOG1041, falls below 12 months, underscoring the poor prognosis with ICI monotherapy in a less selected, heavily pre-treated patient group. These results highlight that ICIs should be introduced as early as possible in the treatment course to maximise patient benefit.

With respect to treatment tolerability, we observed a total of 88% of the patients in our study to experience AEs with 51% classified as grade 3 or higher event. As established by others, we confirm that AEs represent a considerable burden and result in delayed administration of the drug in 39% of included patients and treatment withdrawal in 16%, with colitis being the most common organ manifestation [4]. Maculopapular rash was the most frequently observed irAE in our series (*n* = 14, 5.4%), followed by hypothyroidism (*n* = 12, 4.6%), hepatotoxicity (*n* = 11, 4.3%) and colitis (*n* = 8, 3.1%). Pneumonitis was observed in four patients (1.5%), all of grade 1 or 2. Comparing our irAE profile to the available real-world series, notable differences emerge. In the pembrolizumab cohort reported by Furuya et al. [8], toxicity appeared considerably more manageable, with only 7% of AEs classified as grade 3 or higher – markedly lower than the 51% grade 3 or higher AE rate observed in our cohort. This discrepancy is likely attributable to both the broader ICI exposure in our series and differences in AE ascertainment and documentation practices between settings. These findings highlight the importance of continuous staff education and proactive, multidisciplinary toxicity management when novel immunotherapeutic agents are introduced into routine clinical practice. Timely recognition and appropriate management of irAEs are essential to prevent life-threatening and irreversible complications, and to minimise unnecessary treatment discontinuations [15].

Real-world data estimate the efficacy of a new treatment regimen in a less selected and more representative patient population compared to phase 3 clinical trials, which often employ strict inclusion criteria. Our study captures experience with multiple ICIs in both combination and monotherapy settings within a centralised health region covering approximately 56% of the Norwegian population. The relatively small sample size and the single-centre design represent inherent limitations. Furthermore, the accuracy and completeness of retrospective CTCAE grading is susceptible to underreporting of AEs in clinical records as our study relies on physician-assessed toxicity and does not incorporate patient-reported outcome measures. Conclusions regarding the patient-perceived burden of treatment-related toxicity and it’s impact on health-related quality of life remain limited.

Response rates to ICI monotherapy remain modest, and better predictive biomarkers are needed. While-PD-L1 status – as assessed by CPS [12] – is currently used to guide patient selection for ICI-chemotherapy combination treatment based on the KEYNOTE-826 data, treatment algorithms for recurrent and metastatic cervical cancer continue to evolve in line with international guideline recommendations [17] based on comparable trials such as EMPOWER-Cervical 1 or ENGOT-Cx10/Beat-CC that demonstrated superior efficacy to chemotherapy irrespective of PD-L1-status [16].

Finally, pragmatic trials and real-world analyses offer opportunities to explore alternative dosing regimens and treatment duration. Preliminary data in non-small cell lung cancer suggest that dose-reduced pembrolizumab may achieve similar efficacy with a median PFS of 6.9 months (95% CI: 6.0–9.8) in the standard dose arm and 7.6 months (95% CI: 5.8–11.3) in the reduced dose arm [18]. Several societies including the Gynecologic Cancer InterGroup are actively encouraging the development of pragmatic trials evaluating ICI de-escalation strategies in gynaecological cancers.

## Conclusions

We observed efficacy outcomes in patients with metastatic or recurrent cervical cancer treated with ICIs that are broadly comparable to those reported in the practice-changing KEYNOTE-826 and EMPOWER-Cervical-1 trials [4, 5]. However, we report a higher proportion of patients experiencing AEs relative to the MITO44 and KGOG1041 series [7, 9]. Comparison with another pembrolizumab cohort [8] further highlights that performance status is a key prognostic factor and that toxicity rates in unselected populations may exceed those seen in more carefully selected real-world series. Taken together, these findings emphasise the critical importance of toxicity mitigation training for clinical staff and the need for early, rigorous diagnostic work-up of suspected irAEs when immunotherapy is implemented in an unselected patient population.

## Supplementary Material



## Data Availability

Data will be provided upon request.
