# Supplementary material for: Oncological outcome and immune-checkpoint-blockade-induced toxicities in patients with cervical cancer – a Norwegian real-world cohort
Source: Acta Oncol. 2026 Jun 1;65:45737. doi: 10.2340/1651-226X.2026.45737 (PMC13234913; doi:10.2340/1651-226X.2026.45737)
Supplement: Supplementary file 1 [file AO-65-45737-s1.pdf]

Supplementary material has been published as submitted. It has not been copyedited, or typeset by Acta Oncologica

	Grade 1	Grade 2	Grade 3	Grade 4	Unknown	Total
Alanine aminotransferase increased	7	0	2	0	0	9
Alkaline phosphatase increased	10	3	2	0	0	15
Allergic reaction	0	0	1	0	0	1
Anemia	0	0	23	0	0	23
Anorectal infection	0	0	1	0	0	1
Arthritis	0	1	0	0	0	1
Aspartate aminotransferase increased	8	1	2	0	0	11
Blood bilirubin increased	1	2	1	0	0	4
Blood corticotrophin decreased	0	1	0	0	0	1
Blood prolactin abnormal	3	2	0	0	0	5
Blurred vision	1	0	0	0	0	1
Colitis	1	4	3	0	0	8
Creatinine increased	10	4	0	0	0	14
Diabetes	1	0	0	0	0	1
Diarrhea	1	1	3	0	0	5
Dry eye	1	0	0	0	0	1
Dyspnea	0	0	1	0	0	1
Esophageal infection	0	1	0	0	0	1
Fatigue	0	1	2	0	0	3
Febrile neutropenia	0	0	9	0	0	9
Fever	1	0	0	0	1	2
Gastritis	0	0	1	0	0	1
GGT increased	8	2	0	0	0	10
Headache	0	1	0	0	0	1
Hyperglycemia	1	0	0	0	0	1
Hypertension	0	0	1	1	0	2
Hyperthyroidism	5	2	0	0	0	7
Hypomagnesemia	11	4	0	0	0	15
Hypothyroidism	6	6	0	0	0	12
Infections and infestations - Other, specify	0	0	1	0	0	1
Investigations - Other, specify	4	1	0	0	0	5

**Kidney infection**	**0**	**0**	**1**	**0**	**0**	**1**
**Lung infection**	**0**	**0**	**1**	**0**	**0**	**1**
**Neuralgia**	**0**	**0**	**1**	**0**	**0**	**1**
**Neutrophil count decreased**	**0**	**2**	**1**	**0**	**0**	**3**
**Pain**	**0**	**1**	**0**	**0**	**0**	**1**
**Peripheral sensory neuropathy**	**0**	**2**	**0**	**0**	**0**	**2**
**Pharyngitis**	**0**	**2**	**0**	**0**	**0**	**2**
**Platelet count decreased**	**9**	**6**	**7**	**3**	**0**	**25**
**Pneumonitis**	**1**	**3**	**0**	**0**	**0**	**4**
**Proteinuria**	**0**	**2**	**0**	**0**	**0**	**2**
**Pruritus**	**1**	**0**	**0**	**0**	**0**	**1**
**Psychosis**	**0**	**0**	**0**	**1**	**0**	**1**
**Rash maculo-papular**	**5**	**6**	**2**	**0**	**0**	**13**
**Renal and urinary disorders - Other, specify**	**0**	**1**	**0**	**0**	**0**	**1**
**Sepsis**	**0**	**0**	**4**	**0**	**0**	**4**
**Skin and subcutaneous tissue disorders - Other, specify**	**0**	**2**	**0**	**0**	**0**	**2**
**Thromboembolic event**	**0**	**0**	**4**	**0**	**0**	**4**
**Thyroid stimulating hormone increased**	**5**	**0**	**0**	**0**	**0**	**5**
**Urinary fistula**	**0**	**0**	**1**	**0**	**0**	**1**
**Urinary tract infection**	**0**	**7**	**2**	**0**	**0**	**9**
**Uterine infection**	**0**	**0**	**1**	**0**	**0**	**1**
**Vaginal hemorrhage**	**0**	**0**	**3**	**0**	**0**	**3**
**Total**	**101**	**70**	**81**	**5**	**1**	**258**

Supplementary Table 1: List over all documented AEs in the patient series. Note that patients presenting with elevated TSH only were coded as "Thyroid stimulating hormone increased", while patients with deviating levels of T4 were included under "hypothyroidism".

Supplementary Table 2: Overview over irAEs in the population. Note that individual patients can have experienced more than one irAE · \ Displaying percentage relative to all adverse events (n=258)

ADVERSE EVENT	Grade 1	Grade 2	Grade 3	Grade 4	No (%*)
Maculopapular rash	5	6	3	0	14 (5,4%)
Hypothyroidism	6	6	0	0	12 (4,6%)
Hepatotoxicity	8	1	2	0	11 (4,3%)
Colitis	1	4	3	0	8 (3,1%)
Hyperthyroidism	5	2	0	0	7 (2,7%)
Cholangitis	1	3	2	0	6 (2,3%)
Pneumonitis	1	3	0	0	4 (1,5%)
Arthritis	0	1	0	0	1 (0,4%)
Gastritis	0	0	1	0	1 (0,4%)
Hypophysitis	0	1	0	0	1 (0,4%)
